# Improved scan efficiency for golden-angle radial CMR with anisotropic field-of-view

**DOI:** 10.1186/1532-429X-18-S1-O108

**Published:** 2016-01-27

**Authors:** Ziyue Wu, Fei Han, Peng Hu, Krishna S Nayak

**Affiliations:** 1 grid.42505.360000000121566853University of Southern California, Los angeles, CA USA; 2 grid.19006.3e0000000096326718University of California, Los Angeles, Los Angeles, CA USA

## Background

Radial sampling is often used in dynamic CMR because it is robust to flow and motion, supports short echo times, and provides a diffuse aliasing pattern. One drawback is that current implementations do not support anisotropic field-of-view (FOV). Larson *et al.* [1] previously provided a simple scheme for supporting anisotropic FOV in static radial imaging. We extend the approach to golden-angle (GA) radial imaging [2], and demonstrate improved scan efficiency for real-time cine CMR applications.

## Methods

In conventional GA radial CMR, spoke angles are incremented by 111.25°, which leads to an almost uniform sampling distribution and isotropic FOV for arbitrary temporal windows. When an anisotropic FOV of any convex shape is desired, it can be expressed as a function of the azimuthal angle FOV(Θ). Since the spoke density f(Θ)∝FOV(Θ+π/2), the revised GA scheme should maintain f(Θ) corresponding to the given FOV(Θ) for arbitrary temporal window. Now consider an angle-normalized space where the angles Θ'=T{Θ} and f(Θ')=1, Θ∈[0,π), Θ'∈[0,1). In this space, Θ'[i] is calculated by conventional GA for i^th^ spoke. It is then transformed back to the physical k-space to get the actual azimuthal angle using Θ[i]=T{Θ'[i]}. Based on the inverse transform sampling, if Θ' is uniformly distributed on [0,1), then Θ=F^-1^(Θ') follows distribution F, where F is the cumulative distribution function of Θ. Therefore T{Θ'[i]}=F^-1^(Θ'[i]). In general, T does not have an analytical solution but can be solved with a piecewise linear approximation to F^-1^ from a fully sampled anisotropic FOV pattern calculated by Larson's method. The interpolation was implemented on scanner in real time and the calculation time was negligible. Code is available for download (*mrel.usc.edu/share.html*). Phantom and real-time horizontal long-axis cardiac images were acquired to verify the improved sampling efficiency compared to conventional GA. Gridding was used to reconstruct all images.

## Results

**Figure 1:**
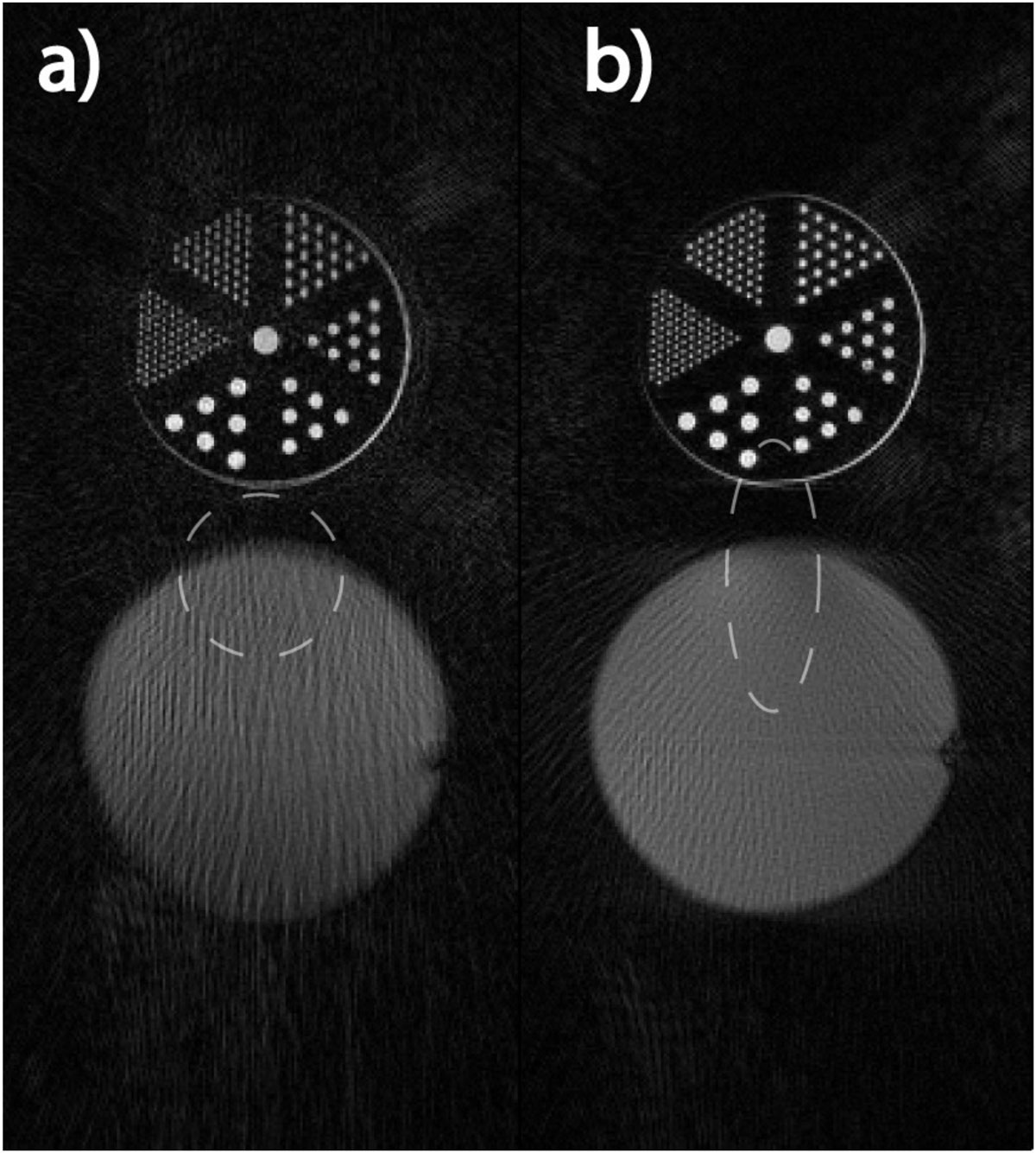
**Phantom images**. Parameters: 144 spokes, 0.75 mm isotropic resolution, 400 × 120 pixels. a) Conventional GA sampling, b) proposed GA sampling for elliptical FOV (major-to-minor-axis ratio 1:0.3). Note the significantly reduced streaking artifacts in b). The unaliased FOV shapes are also shown (white dashed lines) for each sampling scheme.

**Figure 2:**
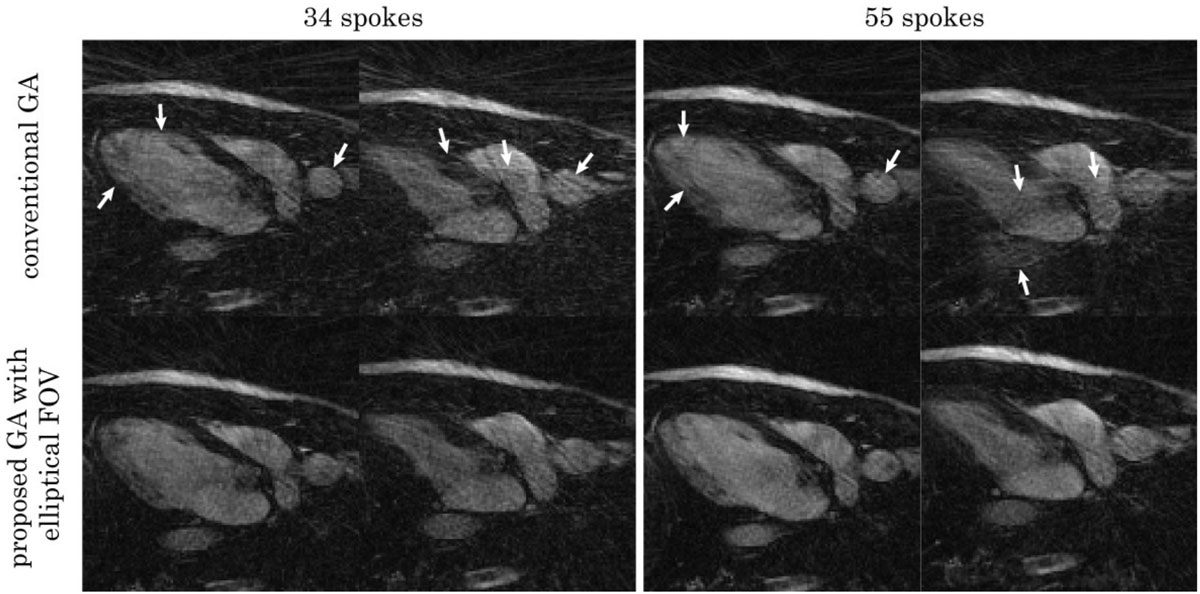
**Horizontal long-axis CMR images from real-time acquisitions during a breath-hold**. Top: diastolic and systolic frames reconstructed with 34 and 55 spokes respectively using conventional GA. Bottom: corresponding frames using the proposed GA with elliptical FOV (major-to-minor-axis ratio 1:0.4). The conventional GA images contain a visibly larger amount of streaking artifacts (white arrows).

Fig. 1 contains images of a ball phantom and an ultra-fine resolution phantom placed side-by-side and acquired using conventional GA and the proposed method with elliptical FOV (major-to-minor-axis ratio 1:0.3). Both images were reconstructed with 144 spokes. Fig. 2 shows representative systolic and diastolic frames reconstructed with 34 and 55 spokes respectively using conventional GA and the proposed method with elliptical FOV (major-to-minor-axis ratio 1:0.4). Note the reduced streaking artifacts in both figures.

## Conclusions

We demonstrate a simple method that improves the efficiency of GA radial CMR imaging, which is particularly useful for real-time cine applications. Phantom and in vivo results confirm that less streaking can be observed with the proposed method after gridding. Because less aliasing needs to be resolved compared to conventional GA, a higher acceleration factor can be expected after combining with parallel imaging and/or constrained reconstruction.
